# Biological Roles of Ornithine Aminotransferase (OAT) in Plant Stress Tolerance: Present Progress and Future Perspectives

**DOI:** 10.3390/ijms19113681

**Published:** 2018-11-21

**Authors:** Alia Anwar, Maoyun She, Ke Wang, Bisma Riaz, Xingguo Ye

**Affiliations:** 1 Institute of Crop Science, Chinese Academy of Agricultural Sciences, Beijing 100081, China; aliaanwar186@gmail.com (A.A.); wangke03@caas.cn (K.W.); bisma.riaz75@gmail.com (B.R.); 2 School of Veterinary and Life Sciences, Murdoch University, WA 6150, Australia; m.she@murdoch.edu.au

**Keywords:** ornithine aminotransferase, drought, salinity, pathogens, proline, arginine

## Abstract

Plant tolerance to biotic and abiotic stresses is complicated by interactions between different stresses. Maintaining crop yield under abiotic stresses is the most daunting challenge for breeding resilient crop varieties. In response to environmental stresses, plants produce several metabolites, such as proline (Pro), polyamines (PAs), asparagine, serine, carbohydrates including glucose and fructose, and pools of antioxidant reactive oxygen species. Among these metabolites, Pro has long been known to accumulate in cells and to be closely related to drought, salt, and pathogen resistance. Pyrroline-5-carboxylate (P5C) is a common intermediate of Pro synthesis and metabolism that is produced by ornithine aminotransferase (OAT), an enzyme that functions in an alternative Pro metabolic pathway in the mitochondria under stress conditions. OAT is highly conserved and, to date, has been found in all prokaryotic and eukaryotic organisms. In addition, ornithine (Orn) and arginine (Arg) are both precursors of PAs, which confer plant resistance to drought and salt stresses. OAT is localized in the cytosol in prokaryotes and fungi, while OAT is localized in the mitochondria in higher plants. We have comprehensively reviewed the research on Orn, Arg, and Pro metabolism in plants, as all these compounds allow plants to tolerate different kinds of stresses.

## 1. Introduction

In nature, plants are simultaneously exposed to a combination of biotic and abiotic stresses, and this severely limits crop productivity worldwide [1,2]. Among abiotic stresses, drought and salinity have the largest effect on crop yield, posing a great challenge to agricultural researchers and plant breeders. It is presumed that by 2025, 65% of the world’s population will live in water-stressed environments and more than 50% of arable lands will become saline [3,4]. Biotic stress, including disease-causing pathogens, also reduces crop yield [5]. As long as a plant is subjected to either biotic or abiotic stress conditions, its final yield will undoubtedly be affected. Therefore, improving crop tolerance to combined stress is urgently needed even though it is the most daunting challenge faced by breeders.

Biotic and abiotic stresses are linked together, as abiotic stress conditions such as drought, salinity, and low-temperature influence the occurrence and spread of pathogens and diseases [6,7,8]. In addition, plants subjected to combined stress show common as well as unique responses depending on the nature of the stress [9,10,11].

Plants exhibit various morphological, physiological, biochemical, and molecular responses to tackle biotic and abiotic stresses. At the physiological and molecular levels, plants respond in similar ways to different abiotic stresses. For example, plants have common drought and salinity tolerance mechanisms because both stresses alter redox homeostasis by disrupting essential metabolic processes [12]. Common physiological responses include changes in leaf number, leaf dry matter content, stomatal density and index, transpiration rate, photosynthetic efficiency, abscisic acid (ABA) content, Na^+^ and K^+^ uptake, proline (Pro) content, and lipid peroxidation [13,14,15,16]. In addition, combined biotic and abiotic stresses have been found to trigger a complex regulatory network of genes, including those involved in Pro and polyamine (PA) synthesis, indicating that they are important for responses to multiple stresses. Thus, it is very important to dissect the functions of these candidate genes involved in drought and salinity tolerance pathways. Because Pro accumulation is a common response to both abiotic and biotic stress, in this review, we specifically focus on the genes involved in Pro metabolism. Under stress (biotic, abiotic, or both) conditions, Pro is synthesized via the glutamate (Glu) pathway or ornithine (Orn) pathway. In the Orn pathway, Pro biosynthesis from Orn is catalyzed by ornithine aminotransferase (EC 2.6.1.13; OAT). OAT is widely present in all organisms and participates in stress-induced Pro accumulation in the cytoplasm [17]. The mechanism of Pro accumulation via OAT under a broad spectrum of stress conditions is not fully understood. Here, we provide a summary of the recent progress in understanding the role of OAT in different organisms with a special focus on plant tolerances to stress, including biotic (pathogens) and abiotic (drought and salinity) stresses.

## 2. Universality of OAT

### 2.1. General Kinetic Properties of the OAT Enzyme

Ornithine aminotransferase (EC:2.6.1.13) alternatively known as ornithine delta aminotransferase (δOAT) is a pyridoxal phosphate (PLP)-dependent enzyme involved in the conversion of Orn into glutamyl-5-semi-aldehyde (GSA) and vice versa, using α-ketoglutarate (αKG) and glutamate (Glu) as co-substrates [18]. Experimentally, this reaction is reversible and can be written as K = [Glu][GSA]/[Orn][αKG]. Here, K is the equilibrium constant, which lies between 50 and 70 at 25 °C. Depending on the amount of substrate, OAT can catalyze the reaction in either direction. This is the key feature indicating that OAT is present at the crossroads of multiple metabolic pathways [19]. The GSA produced in this reaction is not stable, so it is in equilibrium with its more chemically stable cyclic form, pyrroline-5-carboxylate (P5C).

### 2.2. Basic Similarities and Differences among Prokaryotes and Eukaryotes

**Figure 1:**
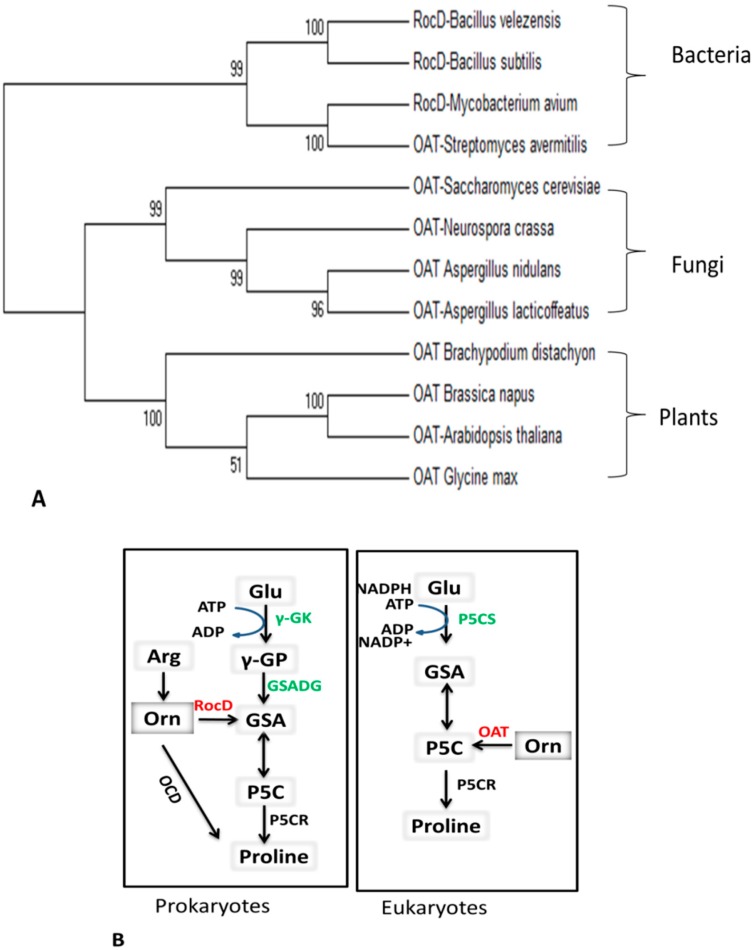
Conservation of the ornithine aminotransferase (OAT) enzyme among prokaryotes and eukaryotes: (**A**) Maximum likelihood phylogenetic tree showing the conservation of OAT enzymes from prokaryotes to higher plants. The tree was constructed using MEGA 6 software with the bootstrap method. Accession numbers of the species used in the study are as follows: *Bacillus subtilis* (NP-391914.1), *Streptomyces avermitilis* (Q82HT8), *Bacillus velezensis* (ABS76054.1), *Mycobacterium avium* (AAS04411.1), *Aspergillus nidulans* (Q92413), *Saccharomyces cerevisiae* (P07991), *Neurospora crassa* (Q7RX93), *Aspergillus lacticoffeatus* (XP_025460070), *Arabidopsis thaliana* (OAO92185), *Brassica napus* (NP_001303219.1), *Glycine max* (XP_003531161.1), and *Brachypodium distachyon* (KQK13994.1). (**B**) Differences in the Glu pathway of Pro synthesis among prokaryotic and eukaryotic organisms. *γ*-GK: *γ*-glutamyl-kinase; *γ*-Glu: *γ*-glutamyl-phosphate; GSADH: glutamic-*γ*-semi-aldehyde dehydrogenase.

OAT is a highly conserved enzyme found in species ranging from prokaryotic bacteria to eukaryotic plants (Figure 1A). Pro synthesis from Glu was first characterized in bacteria and hypothesized to be similar in other prokaryotes and eukaryotes [20,21,22]. Pro synthesis via the Glu pathway starts with ATP-dependent phosphorylation of *γ*-Glu, which is converted into *γ*-glutamyl phosphate (*γ*-GP), then reduced to GSA and spontaneously cyclized to P5C. P5C is a common intermediate of Pro biosynthesis and catabolism (Figure 1B). The Glu pathway was also hypothesized to exist in eukaryotes and higher plants [23]. However, subsequent cloning and characterization of bi-functional pyrroline-5-carboxylate synthase (P5CS) enzymes challenged this hypothesis and revealed the divergence of Pro biosynthesis pathways in other eukaryotes and higher plants [24]. In addition, there is feedback inhibition of plant P5CS by Pro [25]. Similar feedback inhibition was observed for bacterial *γ-*glutamyl-kinase (*γ-*GK) with respect to Glu [26]. The last step of Pro biosynthesis from Glu, which is the reduction of P5C into Pro by pyrroline-5-carboxylate reductase (P5CR), is similar in both prokaryotes and eukaryotes.

In higher plants, there is another route for Pro biosynthesis via the Orn pathway (see Section 3). In prokaryotes (e.g., *Agrobacterium* spp.) Pro can directly be synthesized from Orn by ornithine cyclodeaminase (OCD) or by RocD, which is the OAT enzyme found in bacteria [27]. The bacterial *rocD* gene is involved in the synthesis of Pro via the arginine (Arg) degradation pathway. Arg metabolism is very complex and has several associated pathways [28,29]. One pathway involved in Pro synthesis is the arginase route. The first step in this pathway is the production of Orn and urea from Arg, which is catalyzed by RocF. In a subsequent reaction, Orn is converted into GSA and P5C. The Orn-to-GSA conversion is catalyzed by the RocD (OAT) enzyme. GSA is spontaneously converted into P5C in a reversible reaction. The intermediate P5C is converted into Pro by pyrroline-5-carboxylate reductase (P5CR). RocA also simultaneously acts on P5C and converts it into Glu. Pro and Glu are the final products of the arginase pathway [30]. The second Arg degradation pathway is the Arg deiminase (ADI) route. The first step of this pathway is the deamination of Arg into citrulline (Cit), and then Cit is converted into Orn [19,31]. Orn is then transported outside of the cell membrane and Arg is transported inside via the Arg-Orn antiporter (ArcD) [29].

Other enzymes closely related to OAT such as *N*-acetylornithine δ-aminotransferase (EC 2.6.1.11; NAcOAT) and *N*-succinylornithine δ-aminotransferase (EC 2.6.1.81, SOAT) are also involved in the Arg metabolism pathway and also act on Orn. However, their existence in *Archaeobacteria* is still under debate as they have only been detected by genome analysis [30]. Evidence for the existence of OAT in the roc operon has been reported to be unconvincing as *Mycobacterium tuberculosis* (and other species causing tuberculosis) has a non-functional *rocD* gene [30].

Fungi and higher plants share the pathway for Pro biosynthesis from Arg. In fungi, OAT was first reported in *Neurospora crassa* [32]. In contrast to other eukaryotes where OAT is present in mitochondria, in fungi it is localized only in the cytosol. Localization in the cytosol has been confirmed in *Neurospora* [33], *Saccharomyces cerevisiae* strain X1278b [34], *Agaricus bisporus* [17,35], and *Saccharomycetae* [17,36]. OAT is functionally conserved among fungi and plants [37], and OAT functions are thoroughly described in Section 3.

Unlike in fungi, OAT is a mitochondrial enzyme in plants. OAT is a transaminase involved in the conversion of Orn to GSA. Five decades ago, Orn was first identified in spinach (*Spinacia oleracea)* and mung bean (*Phaseolus aureus*) [38,39]. Then, OAT was partially purified from peanut (*Arachis hypogea*) [40], pumpkin (*Cucurbita maxima*) [41], and squash (*Cucurbita pepo*) [42]. The mitochondrial localization of OAT was revealed by several studies [43,44] and further confirmed by analysis of a GFP-OAT fusion protein [45]. In the late 1990s, several *OAT* cDNA sequences were isolated and functionally characterized in plant species such as *Vigna aconitifolia* [43] and *Arabidopsis thaliana* [44]. Now *OAT* sequences for a number of crop species, including columbine (*Aquilegia)*, barley (*Hordeum vulgare*), alfalfa (*Medicago sativa*), grape (*Vitis vinifera*), maize (*Zea mays*), pine (*Pinus*), potato (*Solanum tuberosum*), rice (*Orzya sativa*), sorghum (*Sorghum bicolor*), and soybean (*Glycine max*), are available in the NCBI unigene and uniProtKB public protein databases (Table 1).

## 3. OAT is Linked with Multiple Metabolic Pathways

### 3.1. OAT and the Pro Metabolic Pathway

**Figure 2:**
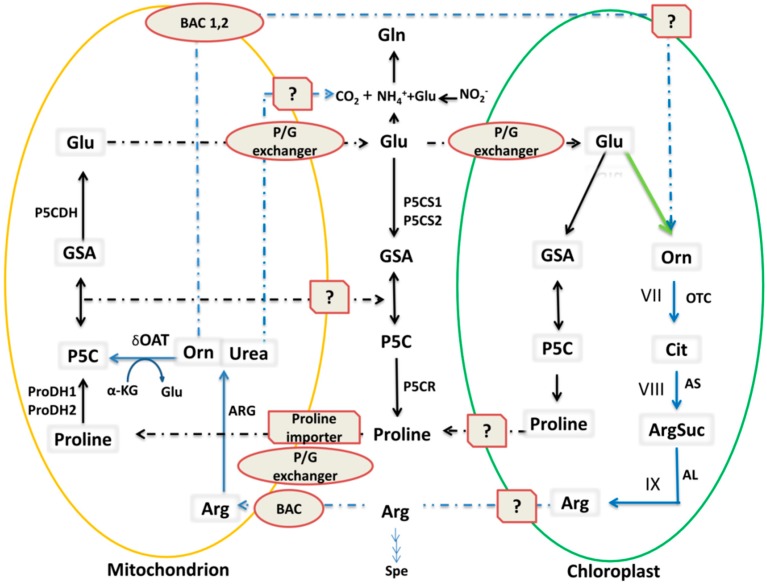
Proline, ornithine, and arginine metabolism and transport in plants. An illustration of the components of the proline (Pro) and arginine (Arg) metabolic pathways that have been identified to date. Data were taken from previously published papers [53,54,55,56,57,58]. Most data were obtained from the model plant *A. thaliana*, but it is hypothesized that this pathway is the same in related plant species. The Pro metabolic pathway is depicted by black lines, the blue lines show the Arg pathway, and the green line shows the ornithine (Orn) pathway, which is further described in Figure 3. Solid lines show cellular pathways while the dotted lines show the intracellular transport of metabolic products. Enzyme transporter proteins are depicted in blue. Glu: glutamate; Arg: arginine; Orn: ornithine; Gln: glutamine; ARG: arginase; Cit: citrulline; OTC: ornithine transcarbamylase; AS: arginosuccinate synthetase; AL: arginosuccinate lyase; ProDH: Pro dehydrogenase; Spe: spermidine; BAC: basic amino acid transporter involved in Arg and Orn exchange; ?: predicted transporters.

The Pro metabolic pathway is involved in Pro biosynthesis and catabolism. In plants, Pro biosynthesis occurs by two pathways, viz. the Glu and Orn pathways. Plants have two isoenzymes that can catalyze the first specific reaction of Pro synthesis: P5CS1 and P5CS2 [46]. In the main Pro biosynthesis pathway, Glu in the cytosol is converted to GSA/P5C (Figure 1) [24,47], which is a common intermediate of Pro biosynthesis and catabolism. Pro biosynthesis from Glu mainly occurs in the cytosol and chloroplast via two enzymatic steps (catalyzed by P5CS and P5CR). Pro catabolism to Glu occurs in mitochondria, also via two enzymatic steps: Pro dehydrogenase (ProDH) and P5C dehydrogenase (P5CDH) catalysis. In the cytosol, the P5C intermediate is reduced into Pro by P5CR [48,49]. Finally, Pro is transported from the cytosol into mitochondria via the mitochondrial Pro/Glu antiporter (P/G) [50]. In mitochondria, Pro is first catabolized into P5C and GSA by the action of ProDH. Then, GSA is converted to Glu via P5CDH, and Glu is transported out of the mitochondria to the cytosol. In this way, Glu is recycled for normal Pro production. Under stress conditions, Pro biosynthesis also occurs in the chloroplast, likely via the same enzymatic steps as in the cytosol, as P5CS1 seems to accumulate in the chloroplast [51,52] (Figure 2).

Besides the Glu pathway in the cytosol, Pro can also be synthesized from Orn via OAT, which is known as the Orn pathway. As a transaminase, OAT transfers the δ-amino group of Orn to α-ketoglutarate, forming GSA and Glu. Experimentally, the equilibrium for this reaction is shifted toward GSA/Glu [22]. GSA is in spontaneous equilibrium with P5C, which is a common intermediate of Pro catabolism and biosynthesis in mitochondria [43,44]. It was hypothesized that formation of GSA/P5C from Orn via OAT constitutes an alternative Pro biosynthesis pathway [43]. The direct contribution of OAT to stress-induced Pro accumulation requires an unknown exit route of GSA/P5C from mitochondria to the cytosol [17]. Although a study conducted in *A. thailana* provides clear evidence for mitochondrial transport of P5C to the cytosol, the identity of the P5C transporter for this route is still unknown [59]. However, several indirect lines of evidence also support this alternative Pro biosynthesis pathway. For example, decreased Pro accumulation is observed in the presence of the OAT inhibitor, gabaculine, in radish cotyledons (*Raphanus sativus*) and detached rice leaves [60,61]. In addition, in *A. thaliana* and *O. sativa*, overexpression of *OAT* enhanced Pro accumulation under salt stress conditions [62,63]. Although the level of Pro accumulation in these transgenic plants was not as high as that in wild type, these studies still indicate that OAT plays a significant role in Pro accumulation under stress. Previously, it was also unclear whether OAT was upregulated in response to stress [43]. However, recently it has been shown that OAT is upregulated three-fold under osmotic stress [64,65], and transcription of the *OAT* gene was found to be regulated by the novel rice stress-responsive NAC (NAM, ATAF, and CUC) transcription factor (TF), SNAC_2_ [66,67]. Some researchers consider OAT and the other stress-regulated enzymes described in Figure 2 to be equally important for conferring resistance against multiple stresses, especially salt stress, because they enhance the synthesis of Pro. The evidence for this comes from studies in which OAT activity was shown to increase under salt stress in radish (*Raphanus raphanistrum*) seeds [17,60] and *A. thaliana* seeds [44] and under oxidative stress in *O. sativa* [66]. All of the above studies provide evidence for an alternative pathway; however, more direct studies are required to confirm Pro biosynthesis via OAT. How Pro functions under stress conditions has recently been reviewed by Liang et al. [68].

### 3.2. OAT is Involved in Arg Catabolism

**Figure 3:**
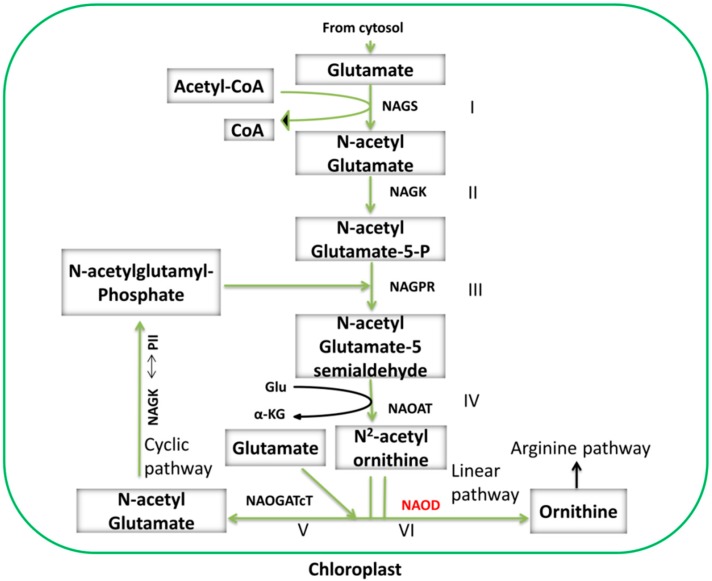
Linear and cyclic ornithine synthesis pathways linked to the arginine metabolic pathway in plants. Arg biosynthesis is divided into two parts and nine discrete steps. In the first part of the pathway, Orn is synthesized from glutamate (Glu), and in the second part, Arg is synthesized from Orn. The first four steps are distinct from the Orn pathway, while the fifth and sixth steps, known as the cyclic and linear pathways, respectively, are also included in the Orn pathway but take different routes. The last three steps (second part) are known as the Arg pathway, which is illustrated in Figure 2. NGS2: *N*-acetylglutamine synthase; NAGK: *N*-acetyl glutamate kinase; NAGPR: *N*-acetylglutamate-5-phosphate reductase; NAOAT: *N*-acetylornithine aminotransferase; NAOGAcT: *N*-acetylornithine-glutamate acetyltransferase; NAGK/PII (a plastid localized protein) double-headed arrow: regulatory interaction between the NAGK and PII proteins; NAOD: *N*-acetylornithine deacetylase.

Arg catabolism begins after Arg is transported into mitochondria by basic amino acid transporter 1 (BAC1) and BAC2. The first step of this pathway is the degradation of Arg into urea and Orn by arginase. Urea is exported into the cytosol where it is converted into ammonia [58] (Figure 2). Here, Orn enters either into the Pro biosynthesis pathway or is transported into the chloroplast where it takes part in the Arg biosynthesis pathway [54,55]. Arg biosynthesis is divided into two parts with a total of nine discrete steps. First, Orn is synthesized through either the linear or cyclic pathways, and then Arg is synthesized from Orn (Figure 3). These steps are described in detail below. The Orn that is used for Arg synthesis may also be exported from the mitochondria via an unknown transporter protein (Figure 2) [54]. Arg synthesis from Orn derived from Glu is well known in plants.

#### 3.2.1. Cyclic and Linear Orn Synthesis Pathways

The conversion of Arg from Glu includes nine discrete steps, and the first four steps are collectively referred to as the Orn pathway or Orn synthesis (Figure 3). Synthesis of Orn from Glu in plants involves several acetylated intermediates [55,69]. Classically, Orn synthesis begins with the formation of *N*-acetyl glutamate from Glu, which is catalyzed by *N*-acetyl glutamate synthase (NAGS) with the help of acetyl-coenzyme A (Acetyl-CoA) [55]. Then, *N*-acetyl glutamate is phosphorylated by *N*-acetyl glutamate kinase (NAGK) to produce N-acetylglutamate-5-P, which is further converted into *N*-acetylglutamate-5-semialdehyde (NAcGSA) in a reaction catalyzed by *N*-acetylglutamate-5-P reductase (NAGPR). In the last step, NAcGSA is converted to *N*-acetylornithine by *N*^2^-acetylornithine aminotransferase (NAOAT), and Orn is released by transfer of the *N*-acetyl group to a Glu residue by *N*-acetylglutamate acetyltransferase (NAOGAcT), a key enzyme allowing the next cycle of Orn synthesis to occur.

The final steps of Orn synthesis are completed via the cyclic or linear pathways. The cyclic Orn synthesis pathway is only found in those organisms that have NAOGAcT, such as non-enteric bacteria, fungi, and plants [70,71,72]. In contrast, some enterobacteria, e.g., *Escherichia coli*, and yeast only have the linear Orn synthesis pathway; these species have *N*-acetylornithine deacetylase (NAOD), which hydrolyzes *N*-acetylornithine to produce Orn [73,74,75]. No NAOD activity has been detected in plants, so the existence of the linear pathway in plants has not yet been confirmed (Figure 3) [55,76,77]. Recently, NAOD activity was revealed in *A. thaliana* through analysis of plants where the *NAOD* gene was inactivated by RNA silencing and T-DNA insertion [78], but further studies are needed to validate the presence of NAOD activity in plants. In chloroplasts, Glu is the precursor of both Orn and Pro, and the destination of Glu is determined by whether it is acetylated or not, i.e., Glu acetylation leads to Orn synthesis and de-acetylation leads to Pro synthesis (Figure 2 and Figure 3) [55,57,79,80].

#### 3.2.2. Synthesis of Arg from Orn

In plants and other organisms, after the formation of Orn, Arg synthesis begins under the control of enzymes in the urea cycle [55,70,81]. Arg is synthesized from Orn via a linear pathway; Orn is first converted into Cit, which is a structural analogue of Arg and accumulates in drought-tolerant plants [72,82,83], by Orn transcarbamoylase (OTC). Cit is further metabolized into Arg by argininosucccinate synthase (AS) and argininosuccinate lyase (AL) (Figure 2) [55,84].

Pro and Arg metabolism are closely associated with OAT levels. Arg is a nitrogen-rich amino acid with a high nitrogen:carbon (4:6) ratio, which makes it suitable for storing nitrogen during senescence and seasonal changes [57]. Arg catabolism is associated with nitrogen remobilization from source tissues, and it also plays a role in developmental processes, especially germination. Catabolism of Arg in mitochondria is the main source of endogenous urea in higher plants, and recycling of urea is very important for plant survival under stress conditions [58,84].

## 4. Biological Roles of OAT

Besides its role in Pro biosynthesis and Arg catabolism, OAT also functions in an alternative pathway for stress-induced Pro accumulation in the cytoplasm, programmed cell death (PCD), and non-host disease resistance in plants [85].

### 4.1. OAT is Involved in Stress-Induced Pro Accumulation

**Figure 4:**
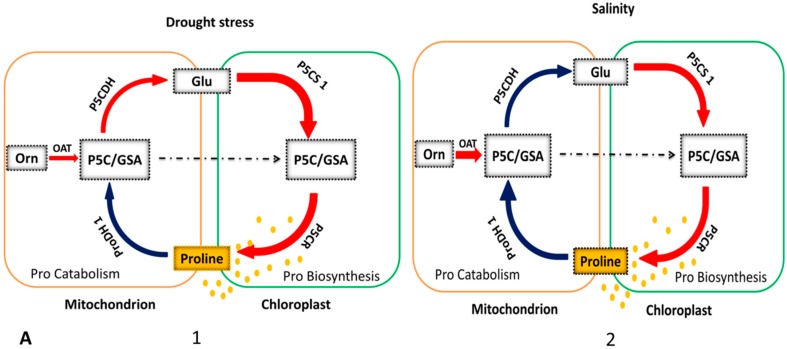

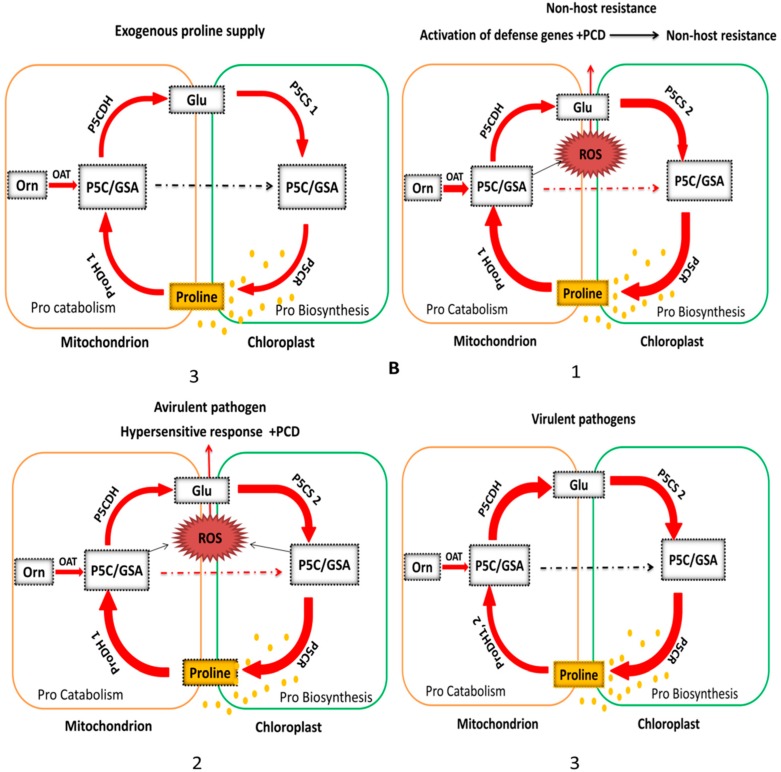
Model for the activation of stress-related genes under different stress environments in plants. There are two groups of stresses: (**A**) abiotic and (**B**) biotic. This diagram shows the up-regulation and down-regulation of the OAT, pyrroline-5-carboxylate dehydrogenase (P5CDH), pyrroline-5-carboxylate synthase (P5CS), pyrroline-5-carboxylate reductase (P5CR), and Pro dehydrogenase (ProDH) enzymes under different stress conditions. Red indicates up-regulation, blue indicates down-regulation, and the thickness of each arrow shows the extent of up- or down-regulation. Yellow dots indicate Pro, which accumulates either in the cytosol or chloroplast depending on the nature of stress. Dotted red lines show activation of P5CR mediated Pro biosynthesis while dotted black lines show normal interacellular transportation of P5C/GSA. In A1 and A2, down-regulation of *ProDH1* results in Pro accumulation during drought and salinity stress. During drought stress, all Pro biosynthesis enzymes are up-regulated and the catabolic pathway is down-regulated to favor Pro biosynthesis. During salinity stress, *P5CDH* is down-regulated and *OAT* is up-regulated to increase Pro biosynthesis. In A3 when exogenous Pro is supplied all stress-related genes are up-regulated. In B1 and B2, increased Pro catabolism due to up-regulation of *ProDH* causes the production of reactive oxygen species (ROS), thus activating defense mechanisms during avirulent and non-host pathogen resistance. In B3, induction of *P5CDH* expression by virulent pathogens prevents pyrroline-5-carboxylate (P5C) accumulation in mitochondria, and activation of the *ProDH* gene results in moderate levels of Pro accumulation, reducing cell death during infection. Additionally, *OAT* expression is increased during non-host pathogen resistance, causing increased production of ROS, which in turn activates the hypersensitive response and other defense responses. PCD: programmed cell death.

Under stress conditions, Pro biosynthesis takes place in the cytosol via the Glu pathway, but under prolonged severe stress conditions, the Orn pathway is upregulated (Figure 4A2,B1) [86]. The first evidence of Pro accumulation in plants was reported in wilting perennial rye grass (*Lolium perenne*) [87]. Later, numerous reports confirmed Pro accumulation under various environmental stresses, such as drought in rice [88], oxidative stress in maize (*Zea mays)* [89], salinity stress in *A. thaliana* [90], high levels of UV exposure in rice, mustard (*Brassica juncea*), and mung bean (*Vigna radiata*) [91], heavy metal stress in *Silene vulgaris* [92], and biotic stress in *A. thaliana* [93,94]. The osmo-protective function of Pro was first revealed in bacteria, and Pro accumulation was found to be positively correlated with salt tolerance [95,96].

After the first report of the presence of OAT in several plants, the gene encoding this enzyme has been successfully cloned and functionally characterized in several crops due to the availability of public databases [39]. OAT has commonly been observed to be involved in Pro metabolism under drought stress, and there are several lines of evidence supporting the association of this enzyme with Pro accumulation during osmotic stress. Increased expression of *OAT* was observed in NaCl-treated radish cotyledons [60] and in *A. thaliana* seedlings exposed to 200 mM NaCl [44,62]. Moreover, Roosens et al. [50] showed that transgenic plants overexpressing *OAT* had higher biomass and germination rates than wild-type plants under osmotic stress. Increased OAT activity in salt-stressed cashew plants and Pro accumulation upon Orn application also provide evidence that stress-induced Pro accumulation occurs via the Orn pathway [97]. In addition, *OAT*-overexpressing rice plants showed significantly increased tolerance to oxidative stress [66]. However, some previous studies have reported contradictory findings. For example, when four-week-old *A. thaliana* plants were subjected to salt stress, free Pro increased, but OAT activity was unchanged [44]. Similarly, in moth bean (*V. aconitifolia*), *OAT* levels decreased in response to salt stress [43] but increased when excessive nitrogen was supplied [98]. On the other hand, a different study concluded that OAT is involved in Arg catabolism rather than in Pro production and has no effect on stress-induced Pro accumulation [45]. Therefore, the role of OAT in stress-induced Pro accumulation in plants is under debate and needs to be clarified, and more studies are required to confirm its multiple roles during biotic and abiotic stress.

These conflicting studies make the role of OAT unclear but open a new direction for study. Recently, our group found that in wheat, Arg is not only involved in conferring resistance against stresses such as drought and salinity, but also is potentially involved in resistance against biotic stresses such as powdery mildew [99]. Previous data from other plant species, also provide evidence that OAT is involved in stress-induced Pro accumulation and plays a significant role in the defense against pathogens. The involvement of Pro in Arg catabolism and localization of OAT next to arginase in the Arg catabolic pathway suggests that OAT may have a role in nitrogen recycling as well. A recent study reinforced this hypothesis and clearly demonstrated that *OsOAT* is essential for nitrogen reutilization. In the OsOAT mutant, abnormalities related to nitrogen deficiency were observed. Based on this observation, a model for *OsOAT* regulation of floret development and seed setting rate was proposed [100]. Our group has successfully cloned and characterized wheat arginase genes [99], and the roles of these genes in nitrogen remobilization and abiotic stress are being investigated.

### 4.2. OAT Is Involved in Plant Non-Host Disease Resistance

Several studies have indicated that Pro and P5C metabolism contributes to plant resistance against pathogens [85,101,102,103]. Senthil-Kumar et al. (2012) found that *AtOAT* and *AtProDH1* play roles in non-host disease resistance through effector triggered immunity. During the first stage of non-host pathogen infection, effectors and pathogen-associated molecular patterns from the pathogen are recognized by plants and induce Pro synthesis in the chloroplast and cytosol. Pro is then transported to the mitochondria where the oxidation of Pro into P5C occurs. Simultaneously, OAT converts Orn into P5C, thereby increasing the level of P5C. Here, P5C takes two routes leading to Pro synthesis: P5CR-mediated Pro synthesis in the cytosol (Figure 2 and Figure 4B1) and P5CS-P5CR-mediated Pro synthesis after conversion to Glu in the cytosol/chloroplast (Figure 2). Both P5C- and ProDH-mediated Pro oxidation can generate reactive oxygen species (ROS) and initiate PCD and the hypersensitive response (HR) and subsequently activate defense signaling pathways (Figure 4B1,B2). In short, non-host resistance, such as that involving OAT, confers immunity to all races of a potential pathogen [104]. Pro also plays a significant role in redox buffering and energy transfer reactions, which lead to plant resistance against pathogens or PCD (Figure 4B2,B3) [52,53,85].

The role of Pro under various oxidative stresses has already been described above. Here, we specifically emphasize the correlation between OAT and Pro with respect to defense against pathogens. Just as Pro accumulates under various abiotic stresses [105,106], Pro also accumulates in *A. thaliana* plants during defense against pathogens [52,93,107]. However, the role of Pro in the defense against pathogen infection has not been fully confirmed because recent studies have shown that Pro catabolism is only enhanced during the early stages of plant infection [108]. Other studies have shown that the intermediate P5C plays a significant role in plant defense against invading pathogens [101,109,110]. The detailed roles of P5C metabolism in plant defense against invading pathogens have been extensively reviewed [85].

The conflicting roles of OAT in Pro metabolism, especially its accumulation in response to virulent and avirulent pathogens, has not yet been clarified. One recent study, in which the *A. thaliana P5CDH* mutant was used to identify possible pathways for Pro synthesis, revealed that *OAT* expression was activated in both mutant and wild-type plants in response to *Pst-AvrRpmI* infection when Pro was supplied exogenously. Orn and Pro levels were also increased [107,111]. Activation of *OAT* under these conditions suggests that Orn is a precursor for Pro synthesis. Increased Orn may be derived from the activation of arginase, which promotes Orn biosynthesis from Arg, as this enzyme is localized in *A. thaliana* tissues infected with *Pst-AvrRpmI* [112]. The requirement for OAT activation for the development of HR in *N. benthamiana* tissues infected with *Pseudomonas syringae pv* provides further evidence for the synthesis of Pro from Orn [53]. The authors of this study thought that the increase in Orn by activation of OAT in the P5CDH mutant resulted in insufficient Pro accumulation. Additional studies are required to formally test this assumption.

### 4.3. Activation of Enzymes Involved in Stress-Induced Pro Accumulation

Enzymes controlling Pro metabolism have been well characterized at both the transcriptional and post-transcriptional levels [113,114]. OAT, P5CS, and ProDH are under transcriptional control, while P5CR and P5CDH are regulated at both the transcriptional and post-transcriptional levels. A gene activation model (Figure 4) shows cyclic up- and down-regulation of these enzymes under multiple stresses. Based on one study of P5CDH mutants, the Pro metabolic pathway was divided into two possible routes: a biosynthetic route (Glu-P5CS-P5C/GSA-P5CR-Pro, Orn-OAT-P5C-P5CR-Pro) and a complete catabolic route (Pro-ProDH-P5C/GSA-P5CDH-Glu) (Figure 3) [111]. In one of these two pathways, Pro biosynthesis from Orn is initiated in mitochondria where OAT mediates transamination of Orn into GSA/P5C [52,68]. In mitochondria, ProDH and OAT activities give rise to the common product, P5C, which is either transformed into Glu by P5CDH, initiating the Glu pathway [45], or transferred into the cytosol where Pro is produced by P5CR [59]. Then, coordination of P5CDH and P5CR activity determines whether P5C is metabolized via the OAT or P5CS Pro biosynthesis pathways under stress [111].

As described above, plants have two isoenzymes that can catalyze the first specific reaction of Pro synthesis: P5CS1 and P5CS2. In most plant species, both isoforms have been identified, but their expression patterns are different under different stress conditions. P5CS1 is up-regulated under osmotic stress (Figure 4A1,A2) [51,90], while P5CS2 is up-regulated during plant pathogen interaction (Figure 4B) [93]. During a stress response, it is most likely that Pro accumulation is due to both up-regulation of Pro biosynthesis and a decrease in Pro degradation. The rate-determining step of Pro degradation is catalyzed by the ProDH enzyme, which has two isoforms: ProDH1 and ProDH 2 [26]. Both isoforms are up-regulated when exogenous Pro is supplied but show different responses to drought and salt stress (Figure 4A) [106,115]. Expression of *ProDH1* is down-regulated during drought and salinity stress [116].

## 5. Future Directions

All available data demonstrate that Pro metabolism has an intricate effect on plant growth and stress responses, and there is no doubt about its osmoprotective function in plant tolerance to abiotic stresses [106,117]. Whether Pro is synthesized via the Glu or Orn pathways, how Pro functions during stress is still under debate because there are two possible mechanisms: (1) The accumulation of Pro, which serves as an osmolyte, via up-regulation of the Pro biosynthesis pathway (Section 4); and (2) the change in Pro metabolic flux during stress, which leads to cell protection by maintaining cellular energy and activation of other signaling pathways that promote cell survival. The underlying molecular mechanisms of how Pro functions during stress are not fully understood, but seem to involve its chemical properties and effects on redox systems. Detailed information on Pro functions under stress conditions has been summarized in previous publications [68,106,118]. All related studies suggest that OAT catalyzes the production of GSA/P5C, which is then converted to Pro by P5CR. Pro production via the Orn pathway is only activated when there is a large amount of nitrogen available or when there is prolonged osmotic stress [45]. Further studies will likely to be focused on understanding how OAT contributes to Pro accumulation under stress conditions in various plant species and whether GSA production from OAT can be directly utilized for Pro synthesis, or if it is necessary for GSA to be first converted into Glu by P5CDH [57]. Therefore, the biological functions of other genes involved in the Orn and Pro pathways, such as *P5CDH*, *P5CS*, *P5CR*, and *ProDH*, also need to be dissected using over-expression and knock-out strategies.

To date, *OAT* has been successfully cloned and functionally characterized in a number of species including potato, pine, grapes, soybean, *A. thaliana*, Medicago, sorghum, barely, maize, and rice (Table 1). However, plant *OAT* genes have not yet been identified in wheat, which is one of the most important cereal crops worldwide with strong drought and salt tolerance. It will be necessary to identify wheat *OAT* genes for the genetic improvement of other economically important plants in terms of resistance to abiotic stresses. Recently, our laboratory has successfully cloned three wheat genes homologous to *OAT* from a wheat express sequence tags (EST) library using a bioinformatics strategy. The three wheat *OAT* genes are located on chromosomes 5AL, 5BL, and 5BL and have complete cDNA sequences that are 1421 bp, 1407 bp, and 1422 bp in length, respectively (unpublished data). The biological roles of the wheat *OAT* genes are being characterized by studying transgenic plants with gain and loss of *OAT* function. The complete functions of the cloned *OAT* genes in other plant species also need to be characterized in more detail.

According to previous studies, the expression of OAT metabolism genes is induced by biotic and abiotic stresses. Presently, a large array of stress-responsive genes has been identified, especially in *A. thaliana* and rice. There are two categories of stress-responsive genes [119]. One category includes functional genes encoding important metabolic enzymes, such as osmo-protective proteins (proline metabolic enzymes), detoxification enzymes, water channels, and late embryogenesis abundant proteins. *OAT*, *P5CDH*, *P5CS*, *P5CR*, and *ProDH* are included in this category. The other category includes regulatory genes such as TFs. Several TF gene families have been reported to be involved in abiotic and biotic stress tolerance in plants, including WRKY, bZIP, MYB, NAC, and AP2/ERF [120,121]. The expression of some members of these families is positively correlated with the expression of the genes encoding proline metabolic enzymes (OAT, P5CDH, P5CS, and P5CR). For example, *SNAC2*, which was identified and cloned in rice, enhances *OAT* expression in transgenic plants [66,122]. Similarly, *ERF1-V* from the AP2/ERF gene family enhances *OAT*, *P5CS*, and *P5CR* expression in wheat [123].

In silico analysis of gene promoter regions has allowed the detection of several putative transcription factor binding sites in stress-responsive genes. In silico analysis of the translation start site of *A. thaliana* genes (*AtOAT, AtP5CS1, AtP5CS2, AtP5CR*) revealed several putative cis-regulatory elements (CREs) recognized by different classes of TFs, including AP2/EREBP, MYB, WRKY, bZIP, and HD-HOX [113]. Similar results were found when putative CREs were investigated in rice [124]. Therefore, CRE analysis could be a useful tool to understand the signal transduction pathways regulating stress responsive genes. However, the results of such in silico analyses need experimental confirmation. Recently, several approaches have been utilized to confirm gene regulatory networks. Yeast one hybrid assays, yeast two hybrid assays, and chromatin immunoprecipitation followed by microarray or sequencing (ChiP-chip and ChiP-seq) and bimolecular fluorescence complementation (BiFC) could be excellent choices to explore the functions of TFs based on protein interactions [125,126].

To date, the biological functions of plant *OAT* genes have mostly been characterized using mutant induction or reverse genetic tools such as interfering RNA (RNAi) gene editing technologies to disrupt gene function have also been successfully developed, including zinc finger nucleases (ZFNs), transcription activator-like effector nucleases (TALENs), and clustered regulatory interspaced short palindromic repeats (CRISPR)/CRISPR-associated protein 9 (Cas9) [127,128]. In particular, CRISPR-Cas9, which can precisely and efficiently edit genes, has been widely used to study target functions through gene silencing [128]. Therefore, CRISPR-Cas9 can be also applied to explore the biological roles of plant *OAT* genes. In fact, our group has successfully edited wheat arginase genes using CRISPR-Cas9, and the edited wheat plants show increased protein content in the grains (unpublished data). The functions of the three wheat *OAT* genes in drought and salt tolerance will be also dissected using CRISPR-Cas9.

## 6. Conclusions

OAT is among the most highly conserved enzymes and is present in species ranging from prokaryotic bacteria to eukaryotic plants. It functions at the crossroads of the Pro, Orn, and Arg metabolic pathways. Under stress conditions, the genes involved in these pathways are activated to combat the stress. Our data linking the Pro, Arg, and Orn metabolic pathways suggest that Orn occupies an important position in the three pathways. There is limited knowledge of the Orn pathway, and this knowledge was mostly gained through research related to Pro and Arg metabolism and, to some extent, polyamine metabolism. The Orn pathway has been dissected by genetic manipulation of OAT in many plant species. *OAT* has been successfully cloned and functionally characterized in many plant species, and genetic manipulation of different plant *OAT* genes demonstrates that it functions as an alternative pathway for stress-induced Pro accumulation. Currently, there is no direct evidence of this alternative Pro pathway. However, numerous studies have provided indirect evidence supporting the existence of this pathway. To further investigate this pathway, we have constructed a gene activation model based on previously published data, which illustrates that OAT is activated during abiotic and biotic stress conditions and is significantly up-regulated during salt stress and non-host disease resistance. More studies on the Orn metabolic pathway are required to help us to understand its exact role in plant stress tolerance.

Arg is considered to be the precursor for Orn synthesis, and OAT converts Orn into Pro as part of one of the two Pro biosynthesis pathways. It has also been concluded that both OAT and Arg are involved in plant resistance to abiotic (drought and salinity) and biotic stress (non-host disease resistance). Nitrogen re-utilization is crucial for the development of new tissues and arginine serves as potential nitrogen source. The presence of OAT next to arginase suggests that OAT also has a potential role in nitrogen re-utilization. In previous investigations, considerable evidence was obtained confirming the role of Pro accumulation under osmotic stress. However, emerging data suggest that TFs are equally important in the expression of Pro biosynthetic genes. Several TF families (WRKY, bZIP, MYB, NAC, and AP2/ERF) have been found to be correlated with the expression of Pro biosynthetic genes. Moreover, in silico analysis has also been performed to identify TFs putatively involved in the regulation of stress responsive gene expression. However, experimental confirmation of these putative TFs is still needed.

## Figures and Tables

**Table 1:** OAT enzymes identified in different plant species.

No.	Name	Accession Number	No.	Name	Accession Number
1	*Arabidopsis thaliana*	OAO92185	20	*Capsella rubella*	XP_006280404
2	*Vitis vinifera*	NP_001268069	21	*Camelina sativa*	XP_010494787
3	*Medicago truncatula*	Q8GUA8	22	*Ricinus communis*	EEF42620
4	*Oryza sativa*	XP_015630389.1	23	*Jatropha curcas*	NP_001306851
5	*Glycine max*	XP_003531161.1	24	*Populus euphratica*	XP_011007419
6	*Sorghum bicolor*	XP_002464174.1	25	*Gossypium hirsutum*	XP_016753478
7	*Zea mays*	NP_001130350.1	26	*Gossypium arboreum*	XP_017641965
8	*Ricinus communis*	XP_002519647.2	27	*Gossypium raimondii*	XP_012450413
9	*Helianthus tuberosus*	AHJ08571.1	28	*Prunus persica*	XP_007214014
10	*Nicotiana attenuata*	XP_019259981.1	29	*Rosa chinensis*	XP_024156782
11	*Brassica napus*	NP_001303219.1	30	*Carica papaya*	XP_021904606
12	*Brassica oleracea*	XP_013593040.1	31	*Cucurbita maxima*	XP_023001421
13	*Brassica rapa*	NP_001288848.1	32	*Solanum tuberosum*	XP_006355410
14	*Hordeum vulgare*	BAJ87243.1	33	*Solanum lycopersicum*	XP_015085834
15	*Aquilegia coerulea*	PIA41644.1	34	*Eutrema salsugineum*	XP_006398303
16	*Nicotiana attenuata*	XP_019259981.1	35	*Cucurbita maxima*	XP_022994797.1
17	*Nicotiana tabacum*	XP_016456334.1	36	*Capsicum chinense*	PHU09018.1
18	*Prunus persica*	ALT55650.1	37	*Capsicum annuum*	XP_016537501.1
19	*Ziziphus jujuba*	XP_009775369.1	38	*Sesamum indicum*	XP_011096597.1

## Abbreviations

ROS	reactive oxygen species
ABA	abscisic acid
OAT	ornithine amino transferase
P5CS	pyrroline-5-carboxylate synthase
P5C	pyrroline-5-carboxylate
P5CR	Pyrroline-5-carboxylate reductase
P5CDH	Pyrroline-5-carboxylate dehydrogenase
ProDH	Proline dehydrogenase
Orn	Ornithine
Glu	Glutamate
PCD	Programmed cell death
GSA	Glutamyl-5-semi-aldehyde
αKG	Alpha ketoglutarate
Pro	Proline
Arg	Arginine
ADI	Arg deiminase
Cit	Citrulline
HR	Hypersensitive
TFs	Transcription factors
